# ChatGPT-Generated Advice on Sun Protection and Skin Cancer Prevention Compared to American Academy of Dermatology Guidelines: Cross-Sectional Content Analysis

**DOI:** 10.2196/93839

**Published:** 2026-06-30

**Authors:** Hanadi Qeyam, Ahmed Al-Rusan

**Affiliations:** 1 Department of Dermatology, Faculty of Medicine, Jordan University of Science and Technology, P.O.Box 3030, Irbid, 22110, Jordan, 962 2 7201000; 2 Birmingham Skin Centre, Department of Dermatology, Sandwell & West Birmingham Hospitals NHS Trust, Birmingham, United Kingdom

**Keywords:** artificial intelligence, AI, skin cancer prevention, sun protection, ChatGPT, dermatology, large language models

## Abstract

**Background:**

Artificial intelligence tools such as ChatGPT are increasingly used by the public to seek health-related information. However, the accuracy and quality of artificial intelligence–generated dermatological guidance, particularly regarding sun protection and skin cancer prevention, have not been systematically assessed.

**Objective:**

This study aimed to evaluate the quality of ChatGPT-generated responses to common patient questions on sun protection and skin cancer prevention by benchmarking them against guidelines from the American Academy of Dermatology.

**Methods:**

Nine standardized questions reflecting common public inquiries were submitted to ChatGPT (GPT-4 free tier) in a single session on May 13, 2025. Responses were independently evaluated by 2 board-certified consultant dermatologists (>15 years’ experience each) across 4 domains (accuracy, completeness, clarity, and relevance) using an author-developed 5-point ordinal rating scale anchored to American Academy of Dermatology guidelines. Scoring disagreements were resolved through discussion between raters until consensus was reached. Interrater reliability was assessed using the linear weighted Cohen κ and intraclass correlation coefficient.

**Results:**

Overall mean scores were 5.0 (SD 0.0) for accuracy (ceiling effect observed), 4.1 (SD 0.6) for completeness, 5.0 (SD 0.0) for clarity (ceiling effect observed), and 4.9 (SD 0.3) for relevance, yielding an overall mean of 4.75/5.0 (SD 0.49). Interrater reliability was excellent (weighted Cohen κ=0.80; intraclass correlation coefficient=0.85; exact agreement on 33/36, 91.7% of the items). Completeness was the lowest-scoring domain (range 3.0-5.0), primarily reflecting errors of omission rather than commission.

**Conclusions:**

ChatGPT provided largely accurate and guideline-consistent advice on sun protection and skin cancer prevention in this targeted content analysis. Its primary limitation was incomplete coverage of nuanced guideline details. While not a replacement for professional health care, ChatGPT may serve as a valuable adjunct tool for public health education on skin cancer prevention provided that its outputs are subject to ongoing, systematic evaluation.

## Introduction

### Epidemiology and Global Burden of Skin Cancer

Although many skin cancers are preventable, there has been a global increase in skin cancer incidence in recent years. Skin cancer accounts for one-third of all cancer diagnoses of the nonmelanoma type, with basal cell carcinomas accounting for 75%. Although melanoma represents only 2% of all skin cancer cases, it is responsible for 80% of skin cancer–related deaths. The annual incidence of melanoma has increased by 4% especially among fair-skinned populations in North America, northern Europe, Australia, and New Zealand [1-3]. In the United States, melanoma is the fifth most common cancer, with an increase in incidence of approximately 320% since 1975 [4]. Globally, melanoma cases are projected to increase from 325,000 in 2020 to approximately 510,000 by 2040—a 50% rise—and mortality is expected to increase by 68% over the same period.

This trajectory underscores the urgent need for effective sun protection strategies and enhanced public health education. UV radiation is the primary modifiable risk factor for skin cancer, causing direct DNA damage to skin cells [5]. Despite growing awareness, adherence to comprehensive sun protection guidelines remains suboptimal [6], creating an education gap that emerging technologies may help address.

### Artificial Intelligence in Patient Education

Given the documented gap between guideline recommendations and public adherence, artificial intelligence (AI) platforms such as ChatGPT represent a new opportunity to improve health communication and patient education at scale. ChatGPT enhances patient education by providing clear and accessible information about dermatological conditions, improving patient understanding and engagement [7]. However, a pilot study by Mondal et al [8] raised significant concerns regarding the accuracy of AI-generated content and potential issues related to text similarity. More broadly, evaluating conversational health AI tools requires frameworks that go beyond surface-level correctness to assess evidence grounding, clinical contextual adequacy, and usefulness to lay users [9].

### Study Aims

This study aimed to evaluate the quality of responses generated by ChatGPT (OpenAI) regarding sun protection and skin cancer prevention by systematically comparing them to American Academy of Dermatology (AAD) guidelines. Using an author-developed assessment framework covering 4 domains—accuracy, completeness, clarity, and relevance—this descriptive content analysis sought to characterize ChatGPT’s utility and limitations as a source of patient-facing dermatological guidance.

The findings will inform patient education strategies and contribute to evidence-based guidelines for AI integration in health care communication. This research is particularly timely given the growing reliance on AI-powered health information resources and their potential impact on public health behaviors.

## Methods

### Study Design

This was a descriptive content analysis comparing AI-generated responses to common patient questions on sun protection and skin cancer prevention against established clinical guidelines. Nine specific questions were developed to cover sunscreen use, protective clothing, and early skin cancer detection, reflecting common public inquiries consistent with topics addressed in previous evaluations of AI-generated dermatological content [10].

A structured summary of the study design following the METRICS (model, evaluation, timing, range/randomization, individual factors, count, and specificity of prompts and language) framework for standardized reporting of generative AI studies in health care [11] is shown in Table 1.

**Table 1.** Model, evaluation, timing, range/randomization, individual factors, count, and specificity of prompts and language (METRICS) summary of study design and reporting parameters.

METRICS component	Definition	This study
Model	AI^a^ model and version evaluated	ChatGPT (GPT-4), free-tier interface; exact subversion not pinnable on the free tier
Evaluation	Approach and instrument used to assess outputs	Independent expert rating by 2 board-certified dermatologists; author-developed 5-point ordinal scale across 4 domains (accuracy, completeness, clarity, and relevance) benchmarked against AAD^b^ guidelines
Timing	Dates of data collection	Single session (May 13, 2025)
Range or randomization	Query selection and randomization	Nine predefined standardized questions on sun protection and skin cancer prevention; no randomization (fixed question set)
Individual factors	Rater characteristics and interrater reliability	Two raters, each with >15 y of experience (based in Jordan and the United Kingdom); weighted Cohen κ=0.80; ICC(2,1)^c^=0.85; 91.7% exact agreement
Count	Number of outputs or data points	One output per question; 9 responses; 36 item-domain ratings
Specificity of prompts and language	Prompt design and language used	Verbatim patient-style questions submitted in English; memory enabled and not reset between questions

a AI: artificial intelligence.

b AAD: American Academy of Dermatology.

c ICC: intraclass correlation coefficient.

### Prompt Development and Question Selection

The following nine questions were presented to ChatGPT: (1) “How to prevent skin cancer?” (2) “What sunscreen should I use?” (3) “How much sunscreen should I use, and how often should I apply it?” (4) “When should I use sunscreen?” (5) “Is a high-number SPF better than a low-number one?” (6) “What to wear to protect my skin from the sun?” (7) “What are the common types of skin cancer?” (8) “How to perform a skin self-exam?” (9) “What are the ABCDE warning signs of melanoma?”

To maintain consistency, questions were submitted verbatim without modifications or additional context, and responses were documented exactly as provided without interpretation. The full responses are shown in Table 2.

**Table 2.** ChatGPT responses to common patient questions on sun protection and skin cancer prevention.

Question	ChatGPT response
1. How to prevent skin cancer?	Preventing skin cancer involves a combination of sun protection, lifestyle habits, and regular monitoring. 1. Protect Your Skin from UV Radiation Use Sunscreen: Apply broad-spectrum sunscreen with SPF 30 or higher every day, even on cloudy days. Reapply every 2 hours and after swimming or sweating. Avoid Peak Sun Hours: Limit direct sun exposure between 10 a.m. and 4 p.m., when UV rays are strongest. Wear Protective Clothing: Use wide-brimmed hats, sunglasses with UV protection, and tightly woven long-sleeved shirts and pants. Seek Shade: Especially when UV levels are high. 2. Avoid Tanning Beds Tanning beds emit harmful UV radiation that increases your risk of all types of skin cancer, including melanoma. 3. Check Your Skin Regularly Perform monthly self-exams to look for new or changing moles or spots. Use the ABCDE rule for melanoma. See a dermatologist annually or if you notice any suspicious changes. 4. Be Aware of Risk Factors Fair skin, a history of sunburns, excessive UV exposure, many moles, and a family history of skin cancer increase your risk. 5. Support Skin Health Stay hydrated and maintain a diet rich in antioxidants to support skin repair and immune defense.
2. What sunscreen should I use?	When choosing a sunscreen, look for the following key features: Broad-Spectrum Protection: Protects against both UVA (aging) and UVB (burning) rays. SPF 30 or Higher: SPF 30 blocks ~97% of UVB rays. Higher SPFs offer slightly more protection but still need reapplication. Water-Resistant: Especially important if you are sweating or swimming. Type of Sunscreen: Mineral (Physical): Uses zinc oxide or titanium dioxide. Good for sensitive skin. Starts working immediately. Chemical: Contains ingredients like avobenzone, octinoxate, or oxybenzone. Lightweight, better for everyday wear. Top Dermatologist-Recommended Sunscreens (as of 2024): EltaMD UV Clear Broad-Spectrum SPF 46 La Roche-Posay Anthelios Melt-in Milk SPF 100 Neutrogena Ultra Sheer Dry-Touch SPF 55 Supergoop! Unseen Sunscreen SPF 40.
3. How much sunscreen should I use, and how often should I apply it?	How Much to Use: For the whole body: About 1 ounce (30 mL), roughly a shot glass full. For the face and neck: About a nickel-sized amount or half a teaspoon. For each arm: About 1 teaspoon. For each leg, front and back of the torso: About 1 teaspoon each. How Often to Apply: Before sun exposure: Apply 15 to 30 minutes before going outside. Reapply every 2 hours, especially if outside for an extended time. Reapply immediately after swimming, sweating heavily, or towel drying. Even water-resistant sunscreen needs reapplication every 40 to 80 minutes when swimming or sweating. Bonus Tips: Use broad-spectrum SPF 30 or higher. Do not forget ears, back of the neck, tops of feet, and scalp/hairline. Check expiration dates.
4. When should I use sunscreen?	You should use sunscreen every day, even on cloudy, cool, or winter days, because up to 80% of UV rays can penetrate clouds. Apply it 15 minutes before going outdoors, and especially when exposed to sunlight during peak UV hours (10 a.m. to 4 p.m.). Sunscreen is also important if you are near water, snow, sand, or concrete, as these surfaces reflect UV rays and increase exposure. Additionally, wear sunscreen indoors if you spend a lot of time near windows, since UVA rays can penetrate glass.
5. Is a high-number SPF^a^ better than a low-number one?	Yes, a high SPF (Sun Protection Factor) offers more protection from UVB rays than a low one, but the difference becomes smaller as the SPF increases: SPF 15 blocks about 93% of UVB rays SPF 30 blocks about 97% SPF 50 blocks about 98% SPF 100 blocks about 99% So while SPF 100 sounds much better than SPF 30, it is only about 1-2% more effective in blocking UVB rays. Higher SPF can be more forgiving if you do not apply enough or reapply as often as needed, but it should not replace good application habits.
6. What to wear to protect my skin from the sun?	UPF Clothing: Choose clothing with a UPF (Ultraviolet Protection Factor) rating of 30 or higher. Long-Sleeve Shirts and Long Pants: Opt for loose-fitting, long-sleeve shirts and long pants made from tightly woven fabrics such as cotton, linen, or UV-protective fabrics. Wide-Brimmed Hat: A brim that is at least 3 inches wide for maximum coverage of face, neck, ears, and scalp. UV-Protective Sunglasses: Sunglasses that block 100% of UVA and UVB rays, labeled “UV400” or “100% UV protection.” Clothing with Mesh or Ventilation: For comfort in hot weather. Scarves or Shawls: For additional neck and chest coverage. Sunscreen on Exposed Skin: Apply broad-spectrum sunscreen with SPF 30 or higher to any exposed skin.
7. What are the common types of skin cancer?	Basal Cell Carcinoma (BCC): The most common type of skin cancer, typically appearing as a small, shiny, or pearly bump or a red, scaly patch. It often occurs on sun-exposed areas like the face, ears, and neck. BCC is slow-growing and rarely spreads. Squamous Cell Carcinoma (SCC): The second most common form, often appearing as a firm, red nodule or a flat lesion with a scaly, crusted surface. More likely to spread than BCC but highly treatable when caught early. Melanoma: The least common but most dangerous type, as it can spread quickly to other organs. It typically appears as an irregularly shaped mole with multiple colors. Early detection is crucial for effective treatment.
8. How to perform a skin self-exam?	Find a Well-Lit Area: Choose a brightly lit room with a full-length mirror and a handheld mirror for hard-to-see areas. Check Your Skin from Head to Toe: Start with your face and work down. Look at your scalp, neck, chest, arms, hands, underarms, back, buttocks, and legs, including the bottoms of your feet. Examine Your Moles and Birthmarks: Look for new growths or irregularities using the ABCDE criteria: Asymmetry, Border irregularity, Color variation, Diameter >6mm, Evolving changes. Look for Other Symptoms: Check for sores that do not heal, bleeding, or itching around a mole. Do Not Forget Your Scalp: Use a comb or blow-dryer to part your hair. Examine Your Nails and Soles of Feet: Look for unusual spots or discoloration. Take Notes: Keep track of any changes and consider taking photos to compare with future self-exams.
9. What are the ABCDE warning signs of melanoma?	A - Asymmetry: One half of the mole or lesion does not match the other half. B - Border: The edges are irregular, notched, or blurry. Normal moles have smooth, well-defined borders. C - Color: The color is uneven, with shades of brown, black, pink, red, white, or blue. D - Diameter: The mole is larger than 6 millimeters (about the size of a pencil eraser), although melanomas can be smaller. E - Evolving: The mole is changing in size, shape, or color over time. Any change should be evaluated by a healthcare professional.

a SPF: sun protection factor.

### Prompt Administration and Reproducibility Conditions

All 9 questions were submitted within a single uninterrupted session on May 13, 2025, using the free-tier ChatGPT interface (GPT-4). The ChatGPT memory function and conversation history were enabled throughout the session and were not reset between questions, meaning that the model retained the full conversational context as subsequent questions were posed. This reflects the typical use pattern of a lay public user and was intentional in capturing the realistic output that a patient would receive in a real-world interaction. However, it represents a methodological limitation affecting response independence as later responses may have been influenced by earlier conversational context (this is discussed further in the Limitations section). The exact model subversion could not be pinned on the free-tier interface; GPT-4 was the version designated to free users at the time of data collection. A single response was collected per question, consistent with a realistic single-query patient use scenario [10].

### Evaluation Framework

ChatGPT’s responses were evaluated against established AAD guidelines using an author-developed 5-point ordinal rating scale covering 4 domains:

- Accuracy—the factual correctness of the statements made, assessed against AAD guidelines and scientific evidence and scored on the correctness of what was stated, not on the completeness of coverage (which was captured separately under the “completeness” domain)
- Completeness—the breadth of coverage; specifically, whether all essential AAD-recommended information points were included in the response
- Clarity—the understandability and accessibility of the information for a lay audience
- Relevance—the practical applicability and directness of the response to the question asked

A 5-point scale was applied to each domain: 0=“completely inadequate or incorrect”; 1=“poor, significant inaccuracies or omissions”; 2=“fair, partially correct with notable gaps”; 3=“good, mostly correct with minor gaps”; 4=“very good, only minor omissions”; and 5=“excellent, fully correct and comprehensive.”

### Raters and Scoring Procedure

Two board-certified consultant dermatologists, each with over 15 years of clinical experience—one based in Jordan (HQ) and one based in the United Kingdom (AA-R)—independently evaluated all ChatGPT responses across the 4 domains. Scoring was completed independently without communication between raters. Score sheets were then compared; where disagreements were identified, the 2 raters discussed each item until consensus was reached without involvement of a third arbitrator. For 2 disagreements (questions 3 and 8 in the “completeness” domain), consensus was reached by averaging scores; for one disagreement (question 5 in the “completeness” domain), rater 2 (AA-R) deferred to rater 1 (HQ) after discussion. Consensus scores are reported throughout.

### Interrater Reliability

Interrater reliability (IRR) was assessed prior to consensus resolution using SPSS Statistics (version 29; IBM Corp). The linear weighted Cohen κ was calculated to account for the ordinal nature of the scale, with weights proportional to the magnitude of disagreement. The intraclass correlation coefficient (ICC) was computed using a 2-way mixed-effects model, absolute agreement, single rater (ICC(2,1)). κ values were interpreted as slight (<0.20), fair (0.21‐0.40), moderate (0.41‐0.60), substantial (0.61‐0.80), and almost perfect (>0.80) agreement. An ICC of 0.75 or higher was considered to indicate excellent reliability [12].

### Statistical Analysis

Descriptive statistics were calculated for each evaluation domain: mean, median, SD, IQR, minimum, and maximum. Given the small sample size (n=9 questions) and ordinal scale, median and IQR values are reported alongside mean and SD values. Domains achieving a mean of 5.0 with an SD of 0.0 reflected ceiling effects and were interpreted accordingly. All analyses were performed using SPSS Statistics (version 29).

### Ethical Considerations

This study did not involve human participants or patient data; therefore, institutional review board approval was not required. As this study involved only the analysis of AI-generated text against published clinical guidelines, no ethical concerns regarding human participants apply. This study was not a randomized clinical trial; accordingly, trial registration and CONSORT (Consolidated Standards of Reporting Trials) reporting requirements do not apply.

## Results

### IRR Results

Preconsensus rating data for all 36 item-domain combinations (9 questions × 4 domains) are shown in Table 3. Raters agreed exactly on 91.7% (33/36) of the items. All 3 disagreements occurred within the “completeness” domain and were of a magnitude of –1 point to +1 point (question 3: rater 1=4 and rater 2=5, resolved by averaging to 4.5; question 5: rater 1=4 and rater 2=3, resolved by rater 2 deferring to rater 1 [score of 4]; and question 8: rater 1=5 and rater 2=4, resolved by averaging to 4.5). No disagreements occurred in the “accuracy,” “clarity,” or “relevance” domains. The linear weighted Cohen κ was 0.80, indicating substantial to almost perfect interrater agreement. The ICC(2,1) was 0.85, confirming excellent reliability.

**Table 3.** Preconsensus rater scores and interrater agreement by domain^a^.

Domain	Rater 1 scores (questions 1-9)	Rater 2 scores (questions 1-9)	Exact agreement, n/N (%)	Disagreements	Resolution
Accuracy	5, 5, 5, 5, 5, 5, 5, 5, 5	5, 5, 5, 5, 5, 5, 5, 5, 5	9/9 (100)	None	—^b^
Completeness	4, 4, 4, 5, 4, 3, 4, 5, 4	4, 4, 5, 5, 3, 3, 4, 4, 4	6/9 (66.7)	Questions 3, 5, and 8 (–1 point to +1 point)	Questions 3 and 8: averaged (score of 4.5); question 5: rater 2 deferred to rater 1 (score of 4)
Clarity	5, 5, 5, 5, 5, 5, 5, 5, 5	5, 5, 5, 5, 5, 5, 5, 5, 5	9/9 (100)	None	—
Relevance	5, 5, 5, 5, 5, 4, 5, 5, 5	5, 5, 5, 5, 5, 4, 5, 5, 5	9/9 (100)	None	—
Overall	—	—	33/36 (91.7)	3 items (3/36, 8.3%)	All resolved within –1 point to +1 point

a Weighted Cohen κ=0.80; intraclass correlation coefficient (ICC(2,1)) 2-way mixed effects, absolute agreement, single rater=0.85*.*

b Not applicable.

### Comparative Analysis of ChatGPT and AAD Recommendations

#### Question 1: How to Prevent Skin Cancer?

ChatGPT’s response was accurate (5.0/5): all preventive measures stated—including sunscreen use, sun avoidance, protective clothing, tanning bed avoidance, and skin self-examination—were factually correct and consistent with AAD guidelines [13]. Completeness obtained a score of 4.0/5; the response omitted wearing sunglasses with UV protection, extra caution near reflective surfaces (water, snow, and sand), shade as a primary prevention method, and specific guidance on protecting children’s skin. These omissions, while not harmful, mean that a patient following only this advice would receive incomplete sun protection guidance. Clarity (5.0) and relevance (5.0) received perfect scores.

#### Question 2: What Sunscreen Should I Use?

The accuracy score was 5.0/5 as the response correctly identified broad-spectrum protection, sun protection factor (SPF) of 30 or higher, water resistance, and mineral vs chemical sunscreen distinctions [14]. Completeness obtained a score of 4.0/5, with omissions including the recommendation to combine sunscreen with other protective measures, extra precautions near reflective surfaces, and guidance on applying an adequate amount. The response included product-specific brand recommendations (EltaMD, La Roche-Posay, Neutrogena, and Supergoop!), which were considered clinically appropriate in context; however, geographic variability in product availability means that such brand-level guidance may not be universally applicable and should be interpreted as illustrative rather than prescriptive [15]. Clarity and relevance scores were both 5.0/5.

#### Question 3: How Much Sunscreen Should I Use, and How Often Should I Apply It?

ChatGPT demonstrated excellent accuracy (5.0/5), correctly stating the standard application amount (1 oz for the full body); the 2-hour reapplication interval; and the need to reapply after water exposure, sweating, or towel drying [14]. The completeness score was 4.5/5, with the only notable omission being lip protection as a specific AAD recommendation. Clarity and relevance scores were both 5.0/5.

#### Question 4: When Should I Use Sunscreen?

ChatGPT received perfect scores across all 4 domains (5.0/5 each). The response covered daily use, weather-related factors, preexposure timing (15 minutes), peak UV hours, high-reflection environments, and indoor application near windows—all consistent with AAD guidelines [14].

#### Question 5: Is a High-Number SPF Better Than a Low-Number One?

The response was clinically accurate (5.0/5), correctly explaining the diminishing marginal protection of higher SPFs and advising against substituting high SPF for good application habits [14]. Completeness obtained a score of 4.0/5, with omissions of the AAD’s minimum SPF 30 recommendation, the fact that a higher SPF does not extend the reapplication interval, and the common problem of underapplication. Clarity and relevance scores were both 5.0/5.

#### Question 6: What to Wear to Protect My Skin From the Sun?

ChatGPT received the lowest scores for this question. Completeness received a score of 3.0/5, with omissions including protective footwear, warnings against ineffective options (baseball caps and straw hats with gaps), the reduced protection of wet clothing, and the substantial variation in UV protection factor (UPF) across fabric types (eg, denim with a UPF of approximately 1700 vs a white T-shirt with a UPF of approximately 7) [16]. Relevance received a score of 4.0/5 because the response did not integrate clothing into the broader multicomponent sun protection strategy. Accuracy was scored with 5.0/5 for the items that were stated; however, it should be noted that the inclusion of “clothing with mesh or ventilation” as a sun-protective option is potentially misleading as mesh and ventilated fabrics typically have lower UPF ratings due to their open weave. This item was not penalized under accuracy because the response did not explicitly claim high UV protection for such garments, but clinicians should note this as a potential point of patient misunderstanding.

#### Question 7: What Are the Common Types of Skin Cancer?

ChatGPT accurately described basal cell carcinoma, squamous cell carcinoma, and melanoma, achieving a score of 5.0/5 for accuracy, clarity, and relevance [17]. Completeness obtained a score of 4.0/5; the notable omission was actinic keratoses, which are clinically important precancerous lesions with progression risk to squamous cell carcinoma.

#### Question 8: How to Perform a Skin Self-Exam?

Accuracy received a score of 5.0/5, with a correct systematic head-to-toe approach and appropriate integration of ABCDE criteria [18]. Completeness received a score of 4.5/5; minor gaps included separate explicit mention of fingernail and toenail examination, examination between the toes, and the AAD’s specific recommendation to consult a board-certified dermatologist when changes are detected. Clarity and relevance scores were both 5.0/5.0.

#### Question 9: What Are the ABCDE Warning Signs of Melanoma?

ChatGPT received perfect scores for accuracy, clarity, and relevance (5.0/5 each), correctly defining all 5 ABCDE criteria consistent with AAD guidelines [19]. Completeness received a score of 4.0/5; the primary clinically meaningful omission was the absence of any recommendation for the patient to document and track moles over time, which the AAD specifically recommends as a key behavior for early detection.

### Quantitative Assessment

ChatGPT’s responses were systematically evaluated using a 5-point ordinal rating scale across 4 domains. Descriptive statistics for each domain are summarized in Table 4, and question-level scores across all four domains are presented in Table 5.

Accuracy and clarity both demonstrated ceiling effects, with all 9 responses receiving scores of 5.0/5 and zero variance (SD 0.0; IQR 0.0). This indicates that, within the scope of the 9 questions evaluated, ChatGPT produced no factually incorrect statements. Relevance also scored highly (median 5.0, IQR 0.0; mean 4.9, SD 0.3), indicating that the responses consistently addressed the question asked with clinically applicable content. Completeness was the weakest domain (median 4.0, IQR 0.5; mean 4.1, SD 0.6; range 3.0‐5.0), with the greatest variability between questions. The lowest-scoring response was that to question 6 (sun-protective clothing; mean 4.25 overall), which also had the lowest completeness score (3.0/5) and the only below-perfect relevance score (4.0/5). Errors across all domains were predominantly omissions rather than factual inaccuracies.

**Table 4.** Descriptive statistics of evaluation criteria scores.

Metric	Accuracy	Completeness	Clarity	Relevance
Mean (SD)	5.0 (0.0)^a^	4.1 (0.6)	5.0 (0.0)^a^	4.9 (0.3)
Median (IQR)	5.0 (5.0-5.0)	4.0 (4.0-4.5)	5.0 (5.0-5.0)	5.0 (5.0-5.0)
Range	5.0-5.0	3.0-5.0	5.0-5.0	4.0-5.0

a The SD of 0.0 for accuracy and clarity reflects a ceiling effect: all 9 responses received the maximum score of 5.0 in these domains.

**Table 5.** Evaluation of ChatGPT’s responses on sun protection and skin cancer prevention compared to American Academy of Dermatology guidelines.

Question	Accuracy score (out of 5)^a^	Completeness score (out of 5)^b^	Clarity score (out of 5)^c^	Relevance score (out of 5)^d^	Overall score, mean (SD)^e^
1. How to prevent skin cancer?	5.0	4.0	5.0	5.0	4.75 (0.50)
2. What sunscreen should I use?	5.0	4.0	5.0	5.0	4.75 (0.50)
3. How much sunscreen should I use, and how often?	5.0	4.5	5.0	5.0	4.88 (0.25)
4. When should I use sunscreen?	5.0	5.0	5.0	5.0	5.00 (0.00)
5. Is a high-number SPF^f^ better than a low-number one?	5.0	4.0	5.0	5.0	4.75 (0.50)
6. What to wear to protect my skin from the sun?	5.0	3.0	5.0	4.0	4.25 (0.96)
7. What are the common types of skin cancer?	5.0	4.0	5.0	5.0	4.75 (0.50)
8. How to perform a skin self-exam?	5.0	4.5	5.0	5.0	4.88 (0.25)
9. What are the ABCDE warning signs of melanoma?	5.0	4.0	5.0	5.0	4.75 (0.50)

a Mean score of 5.0 (SD 0.0) across all questions.

b Mean score of 4.1 (SD 0.6) across all questions.

c Mean score of 5.0 (SD 0.0) across all questions.

d Mean score of 4.9 (SD 0.3) across all questions.

e Mean score of 4.75 across all questions.

f SPF: sun protection factor.

## Discussion

### Summary of Main Findings

This descriptive content analysis demonstrates that ChatGPT (GPT-4) generates largely accurate and guideline-consistent responses to common patient questions on sun protection and skin cancer prevention, with an overall mean score of 4.75/5 against AAD recommendations. The primary area of weakness was completeness (mean 4.1/5, SD 0.6), reflecting a consistent pattern of errors by omission rather than commission. This distinction is clinically meaningful: ChatGPT did not produce factually incorrect information in this analysis but delivered incomplete guidance in most responses, with potential real-world implications for patient behavior that depend on the specific omissions.

### Accuracy and Guideline Alignment

ChatGPT consistently provided accurate advice across all 9 questions, with no factual errors identified. Questions concerning sunscreen use, application frequency, and melanoma warning signs received the highest combined scores, indicating reliable performance on specific, well-defined topics. Questions requiring comprehensive, multicomponent answers—particularly regarding sun-protective clothing (question 6)—received the lowest completeness score (3.0/5) and the lowest overall mean score (4.25/5).

The clinical significance of these completeness gaps should not be underestimated. A patient who receives ChatGPT’s response to question 6 and acts on it exclusively would not be warned that wet clothing provides substantially reduced UV protection, would not know that mesh and ventilated garments may have lower UPF ratings than they appear, and would not receive guidance on protective footwear. For a fair-skinned patient with a high risk profile, such omissions could translate into inadequate sun protection despite perceived compliance. Similarly, the omission of guidance on protecting children’s skin (question 1) is particularly important given that childhood UV exposure is a critical determinant of lifetime melanoma risk.

These findings are consistent with those of prior research demonstrating that ChatGPT provided clinically appropriate recommendations for 96% of core questions in comparable studies, with SPF guidelines and sun-protective clothing among the topics evaluated [10]. The predominance of omission over commission errors in our study aligns with the broader pattern across large language model (LLM) evaluations in clinical medicine, wherein AI-generated content is generally accurate but selectively incomplete.

### Clinical Usefulness and the Accuracy-Completeness Distinction

An important methodological consideration is that surface-level guideline alignment and clinical usefulness are not equivalent. A response may receive a high accuracy score yet remain incomplete, inadequately contextualized, or potentially misleading for patients with specific risk profiles or geographic constraints. Product-specific sunscreen recommendations, for example, may be accurate in the context of widely available products but inaccessible in other health care settings. This distinction—between what is stated being correct and what needs to be stated being complete—should inform how AI-generated health content is evaluated in future research and underscores the importance of using multi-domain evaluation frameworks rather than accuracy alone [9]. Recent work evaluating LLMs for clinical classification tasks has similarly demonstrated that outputs may be technically correct yet clinically insufficient for complex or context-dependent decisions [15].

### AI as an Adjunct to Clinician-Led Education

Previous research has demonstrated that AI-driven interventions such as skin aging simulations have produced long-term improvements in sun protection behaviors, particularly among younger adults [20]. This suggests that AI tools such as ChatGPT could serve not only as information sources but also as platforms that motivate adherence to sun protection recommendations. Consistent with a human-centered framework for AI in health care and education, ChatGPT is most appropriately positioned as an adjunct that supports and extends clinician-led patient education rather than a stand-alone substitute for professional guidance [12]. Patient-facing AI tools are most effective when integrated into a care model that maintains physician oversight and encourages users to seek professional confirmation of AI-generated advice.

While this study focused on preventive education, AI is also being used in clinical dermatology for diagnostic support. A recent review demonstrated that AI can accurately analyze clinical and dermoscopic images, sometimes surpassing human experts in specific settings, but emphasized that the best outcomes require combining AI tools with clinical expertise [21].

### Comparison With Other AI Platforms

This study evaluated ChatGPT (GPT-4) exclusively, which was the most widely available and most extensively studied conversational AI tool for health information at the time of data collection (May 2025). Other platforms—including Google Gemini, Grok, and Anthropic’s Claude—may generate meaningfully different responses to the same prompts and have not been benchmarked against AAD guidelines in this context. Multi-platform comparative evaluation represents an important direction for future research.

### Limitations

Several limitations should be acknowledged. First, the 9 questions constitute a targeted content analysis rather than a comprehensive evaluation of ChatGPT’s dermatological knowledge scope; follow-up questions, ambiguous prompts, and unsafe queries were not assessed. Second, all questions were submitted in a single session, with memory enabled and conversation history not reset between questions, meaning that later responses may have been influenced by prior conversational context. Third, a single output was collected per question; given the stochastic nature of LLMs, responses may vary across sessions, and findings reflect a single–time point snapshot. Fourth, ChatGPT’s outputs are subject to change as underlying model versions are updated, and findings from May 2025 may not be reproduced with future versions. Fifth, 2 raters were used; although IRR was excellent (weighted κ=0.80; ICC=0.85), a larger panel would further strengthen scoring validity. Sixth, the evaluation used an author-developed rating scale without formal psychometric validation; future studies should develop and validate a standardized instrument. Seventh, the comparison was limited to AAD guidelines; international guideline variations were not assessed. Eighth, all prompts were submitted in English; because prompt language has been shown to critically shape the readability, completeness, and terminology of AI-generated dermatological content, responses to equivalent prompts in other languages may differ, and our findings may not generalize to non–English-speaking populations [22]. Finally, product-specific brand recommendations in ChatGPT responses may reflect products not available in all geographic regions.

### Conclusions

This descriptive content analysis demonstrates that ChatGPT generates largely accurate and guideline-consistent advice on sun protection and skin cancer prevention, with responses closely aligned with AAD recommendations across all 4 evaluated domains. Errors were predominantly omissions rather than factual inaccuracies, with completeness representing the primary area for improvement. The clinical significance of these omissions varies by question but warrants attention, particularly for high-risk patient groups. Although ChatGPT cannot replace professional medical advice, it can serve as a valuable adjunct tool for public health education—most effectively when integrated within a clinician-led educational framework that ensures contextual appropriateness and guideline completeness [12]. With appropriate, ongoing evaluation, AI-assisted dermatological education has significant potential to support skin cancer prevention strategies.

## Abbreviations

AAD: American Academy of Dermatology
AI: artificial intelligence
CONSORT: Consolidated Standards of Reporting Trials
ICC: intraclass correlation coefficient
IRR: interrater reliability
LLM: large language model
SPF: sun protection factor
UPF: UV protection factor
